# Transcriptome Analysis of Stem and Globally Comparison with Other Tissues in *Brassica napus*

**DOI:** 10.3389/fpls.2016.01403

**Published:** 2016-09-21

**Authors:** Liyun Miao, Libin Zhang, Nadia Raboanatahiry, Guangyuan Lu, Xuekun Zhang, Jun Xiang, Jianping Gan, Chunhua Fu, Maoteng Li

**Affiliations:** ^1^School of Life Science and Technology, Huazhong University of Science and TechnologyWuhan, China; ^2^Hubei Collaborative Innovation Center for the Characteristic Resources Exploitation of Dabie Mountains, Huanggang Normal UniversityHuanggang, China; ^3^Oil Crops Research Institute, Chinese Academy of Agricultural SciencesWuhan, China

**Keywords:** *Brassica napus*, transcriptome, RNA-sequencing, tissue-specific genes, transcription factors

## Abstract

*Brassica napus* is one of the most important oilseed crops in the world. However, there is currently no enough stem transcriptome information and comparative transcriptome analysis of different tissues, which impedes further functional genomics research on *B. napus*. In this study, the stem transcriptome of *B. napus* was characterized by RNA-seq technology. Approximately 13.4 Gb high-quality clean reads with an average length of 100 bp were generated and used for comparative transcriptome analysis with the existing transcriptome sequencing data of roots, leaves, flower buds, and immature embryos of *B. napus*. All the transcripts were annotated against GO and KEGG databases. The common genes in five tissues, differentially expressed genes (DEGs) of the common genes between stems and other tissues, and tissue-specific genes were detected, and the main biochemical activities and pathways implying the common genes, DEGs and tissue-specific genes were investigated. Accordingly, the common transcription factors (TFs) in the five tissues and tissue-specific TFs were identified, and a TFs-based regulation network between TFs and the target genes involved in ‘Phenylpropanoid biosynthesis’ pathway were constructed to show several important TFs and key nodes in the regulation process. Collectively, this study not only provided an available stem transcriptome resource in *B. napus*, but also revealed valuable comparative transcriptome information of five tissues of *B. napus* for future investigation on specific processes, functions and pathways.

## Introduction

*Brassica napus* as one of the world’s most important oilseed crops provides not only edible oil but also raw materials for livestock feed and biofuel applications (Cai et al., 2015; Wood et al., 2015). The allotetraploid *B. napus* (A_n_A_n_C_n_C_n_, 2*n* = 38) was formed by hybridization between *B.* *rapa* (A_r_A_r_, 2*n* = 20) and *B. oleracea* (C_o_C_o_, 2*n* = 18) (Chalhoub et al., 2014). Due to polyploidy, *B. napus* genomes contain a large amount of repeat and homologous sequences (Cai et al., 2015). Multiple copies of most genes in *B. napus* resulted in generating a highly complex and redundant transcriptome (Parkin et al., 2010), which precluded accurate transcript differentiation (Trick et al., 2009), limited the ability to elucidate the genetic mechanisms controlling a trait (Parkin et al., 2010), and imposed a huge challenge in high-throughput genotyping with sequencing and array technologies (Cai et al., 2015). Unambiguous transcript identification technology is essential for accurate estimation of gene expression in *Brassica* species (Parkin et al., 2010). Next generation sequencing technologies, that can capture millions of long sequence tags, have been proved to revolutionize genetic analysis (Mardis, 2008) and possess the ability to comprehensively catalog gene expression in polyploid species (Parkin et al., 2010).

RNA-seq using next generation sequencing was widely used for comparative transcriptome analysis of *B. napus*. The leaf transcriptome of Tapidor, Ningyou 7, Altasweet, Ceska, and Aphid resistant rape were sequenced, a large number of differentially expressed genes (DEGs) were identified and both immediate and long-term alterations in the expression of homologous gene pairs following polyploidy were confirmed (Higgins et al., 2012). Comparative transcript profiling of the gynoecium development in female sterile *B. napus* was investigated, and the possible metabolic pathways behind the formation of the damaged gynoecium were revealed (Fu et al., 2014). The transcriptome profiles of the leaves and roots of *B. napus* were investigated and a mechanism that responds to sudden increase in salinity was obtained (Yong et al., 2014). The difference of mechanism for lipid biosynthesis in leaves and developing seeds of *B. napus* was revealed using comparative transcriptome analysis (Chen et al., 2015), and the carbohydrate and lipid metabolism blocks in *B. napus* male sterility induced by the monosulfuron ester sodium were also depicted (Li et al., 2015).

Zhongyou 821 (Abbr. ZY821) is one of the most outstanding *B. napus* cultivars with high erucic acid in China (Hu et al., 2009). The transcriptional profiles between ZY821 and a low erucic acid cultivar of Zhongshuang 9 were compared, 32 genes involved in lipid biosynthesis during seed development were analyzed and the mechanism of these genes responded to quality improvement was elucidated (Hu et al., 2009). A total of 18 AFLP markers linked to seed coat color trait were identified using a DH population derived from the F1 plants of ZY821 (black-seeded) and No. 2127-17 (yellow-seeded) (Xiao et al., 2007). The accumulation of phenolic compounds in the seed coats of ZY821 was analyzed by comparison with a yellow-seeded cultivar GH06, and significant differentially expression genes in the flavonoid biosynthetic pathway were revealed (Qu et al., 2013). ZY821 was also used as a resistant control to detect QTLs involved in resistance to *Sclerotinia sclerotiorum* (Zhao and Meng, 2003). Ten of DEGs that encoding defense-associated proteins and transcription factors (TFs) involved in plant defense signal pathways were revealed from comparison between ZY821 and a *S. sclerotiorum* susceptible cultivar N-0-1, by using a *B. napus* oligo-nucleotide microarray (Zhao et al., 2009). Additionally, two NAC genes (*BnNAC2 and BnNAC5*) induced by high-salinity, drought, and abscisic acid were identified in 7-day-old seedlings of ZY821, and *BnNAC2* was revealed to preferentially expressed in flowers, whereas *BnNAC5* accumulated at the highest level in stems (Zhong et al., 2012). The *MSI-99m* gene, a synthesized magainin II analog with inhibitory effects to microbial organisms, was transferred into ZY821 to increase resistance to *S. sclerotiorum* (Sang et al., 2013). However, there was currently not enough stem transcriptome information of ZY821 and comparative transcriptome analysis of different tissues, which limited further functional genomics research on this *B. napus* cultivar.

In this study, the stem transcriptome of ZY821 at initial flowering stage was analyzed by using RNA-Seq technology, and about 13.4 Gb high-quality clean reads were obtained (SRA number: SRX1142564), which then were used for comparative transcriptome analysis with the existing transcriptome sequencing data of roots, leaves, flower buds, and immature embryos of *B. napus*. We discovered the common genes, DEGs between stems and other tissues, and tissue-specific genes in different tissues. In the meanwhile, common TFs in the five tissues and tissue-specific TFs were identified, and a TFs-based regulation network between TFs and the target genes involved in ‘Phenylpropanoid biosynthesis’ pathway was constructed. These results provided a comprehensive genomic resource for gene expression analysis of different tissues from *B. napus.*

## Materials and Methods

### Plant Material and RNA Preparation

ZY821 was cultivated in the experiment field of Huazhong University of Science and Technology (China). The seeds were sown in the end of September, 2012. The middle stems were collected after growth at initial flowering stage on March 5, 2013. The collected stems were immediately frozen in liquid nitrogen. Total RNA was extracted from the stems using TRIzol reagent (Invitrogen, Carlsbad, CA, USA) and purified using RNeasy Plant Mini Kit (Qiagen, Valencia, CA, USA) according to the manufacturer’s instructions. Total RNA was quantified by using a NanoDrop spectrophotometer (Thermo Fisher Scientific, Inc.), and the purity of the total RNA was detected by measuring both the A260/280 and A260/230 ratios. The integrity of the RNA samples was assessed with Bioanalyzer 2100 (Aligent, Santa Clara, CA, USA). The purified RNA was dissolved in RNase-free water and stored at -80°C. About 60 μg of total RNA at a concentration of ≥400 ng/μl, OD260/280 = 1.8–2.2, RNA 28S:18S ≥ 1.0, and RNA integrity number (RIN) ≥ 7.0 were used for cDNA library preparation.

### Preparation of cDNA Library and Sequencing

The TruSeq^TM^ RNA Sample Preparation Kit (Illumina, San Diego, CA, USA) was used to construct cDNA sequencing libraries of the stem transcriptome from ZY821. In brief, poly-A mRNA was firstly purified from the total RNA using poly-T oligo-attached magnetic beads. Subsequently, the purified poly-A mRNA was fragmented into smaller pieces (200–700 bp) using an RNA fragmentation kit. The small fragments mRNAs were used as templates for the synthesis of first-strand cDNA with hexamer primers and SuperScript II reverse transcriptase (Promega), and then DNA polymerase I and RNase H were used to synthesize the second-strand cDNA. The cDNA fragments were then purified, end-repaired, A-tailed, and ligated to index adapters (Illumina). The ligated products were subsequently PCR-amplified to generate cDNA libraries, which were sequenced using the Illumina HiSeq 2000 sequencing platform at the NextOmics Biosciences Institute (Wuhan, China).

### Assembly and Mapping of the Clean Reads

After sequencing of the cDNA library, the raw reads were filtered with NGSQC toolkit (v2.2.3) (Patel and Jain, 2012) with the parameters: library type of ‘paired-end,’ primer/adaptor library of ‘Paired end DNA library,’ cut-off read length for HQ (high-quality) of 70%, cut-off quality score of 20, and only statistics of ‘Off.’ The dirty raw reads including those with adaptors, containing more than 10% of unknown bases, and low quality reads (the percentage of the low quality bases of quality value ≤5 is more than 50% in a read) were discarded to generate high quality clean reads. Subsequently, these clean reads were mapped to *B. napus* reference genome^1^ using Tophat2 (v2.0.13) (Trapnell et al., 2009) with the parameters of 50 min-intron-length (int), 500,000 max-intron-length, 3 max-insertion-length (int) and 3 max-deletion-length (int). The gene coverage was calculated as the number of mapped reads in a locus multiplied by 100 bp and then divided by the summed exon length of the locus. Additionally, the transcriptome raw data of leaves, flower-buds, immature embryos and roots of *B. napus* were downloaded and filtered with NGSQC toolkit (v2.2.3) to generate clean reads, and all of the clean reads were also mapped to the reference genome using Tophat2 (v2.0.13) software.

### Transcriptome Annotation

Gene Ontology (GO) annotation was performed using Web Gene Ontology Annotation Plot (WEGO) software (Ye et al., 2006) to obtain cellular component, molecular function, and biological process terms. GO enrichment analysis applied a hypergeometric test to find significantly enriched GO terms in DEGs comparing to the genome background (Fu et al., 2014), the calculating formula was the same as previously described (Yan et al., 2013), and the GO terms with an adjusted *p*-value of ≤0.05 were defined as significantly enriched GO terms in DEGs.

Kyoto Encyclopedia of Genes and Genomes (KEGG) pathways annotation was performed using BLASTX with an *E*-value threshold of 1.0*E*–5 by mapping the sequences obtained from BLAST2GO (Conesa et al., 2005) to the contents of the KEGG metabolic pathway database (Kanehisa and Goto, 2000) to reveal molecular interaction network and metabolic pathways. Pathway enrichment analysis also applied a hypergeometric test to identify significantly enriched metabolic pathways or signal transduction pathways in DEGs comparing with the whole genome background, using the KEGG database. The calculating formula was the same as that in GO analysis (Yan et al., 2013), the pathways with an adjusted *p*-value of ≤0.05 were defined as significantly enriched KEGG pathways of DEGs.

### Identification of Common Genes, DEGs, and Tissue-Specific Genes

Reads per kilobase of exon model per million mapped reads (RPKM) method was used to calculate the expression levels of transcripts (Mortazavi et al., 2008). RPKM = (total exon reads)/[mapped reads (Millions) × exon length (kilobase)], and the RPKM threshold value was 0.1 in this study. All of the expressed transcripts with RPKM value equal to or more than 0.1 in the five tissues were used for analysis of common genes, differently expressed genes (DEGs) and tissue-specific genes. By mutual comparison among the stem transcripts, root transcripts, leaf transcripts, flower bud transcripts and immature embryo transcripts of *B. napus*, the genes that existed in all of the five tissues were defined as common genes, and the genes that only existed in unique specific tissue were defined as tissue-specific genes. The common genes showing different expression levels were defined as DEGs, and the DEGs between stems and other tissues were evaluated using the expression levels of stem transcripts as control and tested with the software package DESeq2 (version: 1.12.3) (Anders and Huber, 2010; Dillies et al., 2013) with an adjusted *p*-value <0.05 and normalized fold change ≥2.

### qRT-PCR Validation of DEGs

Total RNA samples was extracted from the stems, roots, leaves, flower buds, and immature embryos of ZY821, respectively, and then treated with DNase I (Invitrogen). mRNA expression levels of the DEGs in the five tissues were determined by quantitative reverse transcription-PCR (qRT-PCR). One microgram of total RNA was reverse-transcribed using SuperScript III Reverse Transcriptase (Invitrogen) and oligo (dT)18 according to the manufacturer’s protocol. Gene-specific primers for randomly selected 20 DEGs were designed according to the reference sequences using the oligo 7.0 software, all primer sequences were listed in **Supplementary Table 1**. The primer number ranged from 18 to 25 bp, and the length of the amplified product for genes was limited in the range of 80–200 bp. A primer was also designed for *B. napus ACT 7* gene (Fujisawa et al., 2009) as an internal reference. The qRT-PCR experiment was carried out using an ABI 7500 Real-Time PCR System (ABI) and each reaction was performed in triplicate. The *ACT 7* gene was used as an internal control for data normalization, and quantitative variation in the different replicates was calculated using the delta–delta threshold cycle relative quantification method.

### Identification of Transcription Factors

All of transcripts were searched against the plant transcription factor database (PlnTFDB)^2^ by using Blastx with a cut-off *E*-value of 1.0*E*-10 (Kalra et al., 2013) to identify TFs.

### Network Analysis of Transcription Factors and Genes

The network was constructed and analyzed using Cytoscape-V3.2.0 with the Agilent Literature Search Plug-in (Shannon et al., 2003). Search controls option was set by using the default parameters, including max engine matches of 10, selection of ‘use context’ and ‘concept lexicon restricts search.’ In addition, extraction controls option was set by choosing *Arabidopsis thaliana* in concept lexicon, and choosing ‘relaxed’ in interaction lexicon. The network was laid out by using attribute circle layout.

## Results

### mRNA-Seq of the Stem Transcriptome

Total RNA was extracted from the stems of ZY821 at initial flowering stage, and cDNA libraries were prepared and sequenced with Illumina HiSeq sequencing instrument. As a result, 136,785,766 raw reads were produced, and 134,015,130 clean reads (97.97% of raw reads) (SRA number: SRX1142564) with a mean length of 100 bp were generated after removing adaptor sequences, ambiguous reads and low-quality reads (**Table 1**). The stem clean reads were then mapped to the reference genome of *B. napus Darmor-bzh*^3^ (Chalhoub et al., 2014), and 121,415,423 (90.60%) of the clean reads had perfect match to the reference genome, in which 73.50, 3.19, and 23.31% of them were mapped to exonic regions, intronic regions and intergenic regions, respectively.

**Table 1:** Output and mapping of the clean reads from the tissue transcriptome data of *B. napus*.

Transcriptome sample	Accession number	Clean reads number	Reads length	Clean Q30 bases rate	Mapped reads	Mapping rate
Stems	SRX1142564	134,015,130	100	94.31%	121,415,423	90.60%
Roots	SRX332373	51,401,052	90	89.72%	44,896,620	87.35%
Leaves	ERX014871	18,974,743	80	86.64%	16,731,546	88.15%
Flower buds	SRX332269	6,945,504	49	95.73%	6,457,007	92.97%
Immature embryos	SRX710669	23,760,393	80	92.98%	21,902,085	92.19%

For comparative transcriptome analysis in different tissues, the transcriptome raw data of roots of *B. napus* N119 (SRA number: SRX332373), leaves of *B. napus* Ningyou 7 (SRA number: ERX014871), flower buds of *B. napus* H3 (SRA number: SRX332269), and immature embryos of *B. napus* CR3170 (SRA number: SRX710669) were downloaded. These transcriptome raw data were filtered with NGSQC toolkit (2.2.3) to generate clean reads, respectively (**Table 1**), and then mapped to the reference genome using Tophat2 (v2.0.13) software. As a result, 44,896,620 (87.35%) clean reads of root transcripts, 16,731,546 (88.15%) of leaf transcripts, 6,457,007 (92.97%) of flower bud transcripts and 21,902,085 (92.19%) of immature embryo transcripts had perfect match to the reference genome, respectively (**Table 1**). The expression levels of all transcripts were calculated with RPKM method, and a total of 62,910 stem transcripts, 67,922 root transcripts, 61,828 leaf transcripts, 65,960 flower bud transcripts and 57,849 immature embryo transcripts with RPKM value equal to or more than 0.1 were used for further analysis (**Supplementary Table 2**).

### GO Classification

**FIGURE 1:**
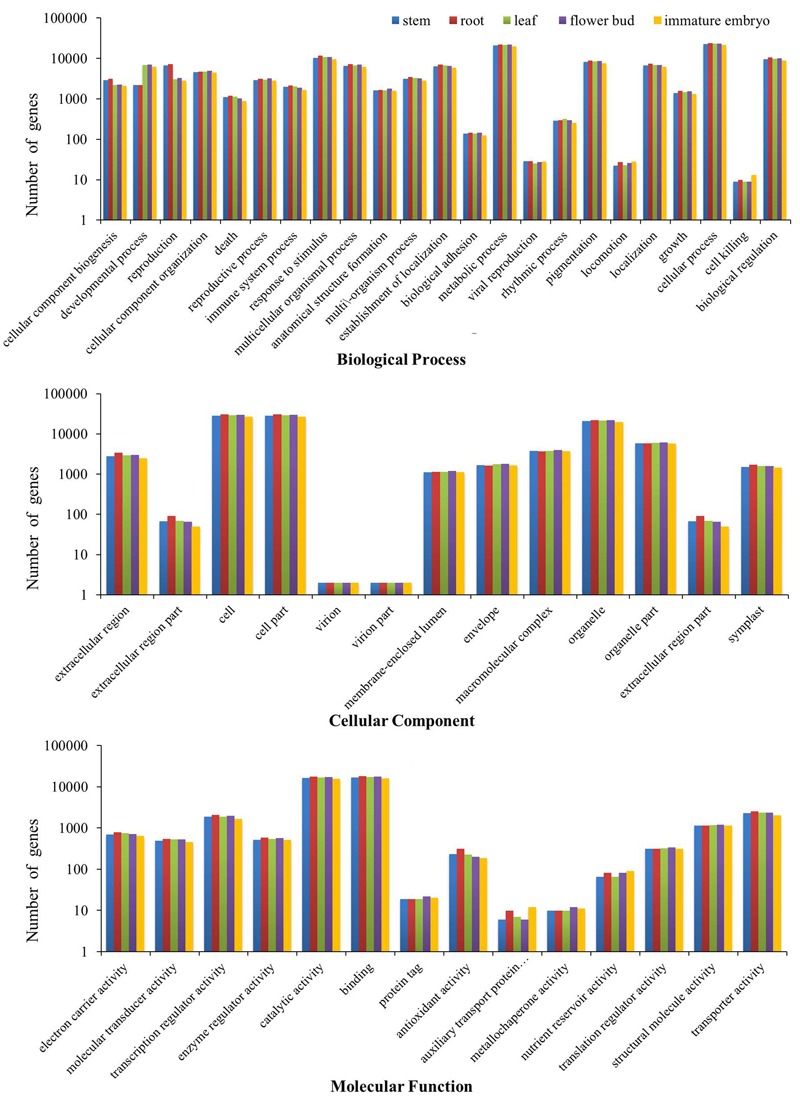
**Three GO categories of the transcripts of the stems, roots, leaves, flower buds, and immature embryos**.

All of the transcripts from the five tissues were functionally categorized by plotted in GO database using WEGO software. As a result, 37,477 stem transcripts, 40,480 root transcripts, 38,656 leaf transcripts, 39,423 flower bud transcripts and 35,088 immature embryo transcripts were annotated by GO databases and categorized in three main GO categories: biological process, cellular component and molecular function, and these were further classified into 50 functional sub-categories (**Figure 1**). In biological process, 28,107 stem transcripts, 30,354 root transcripts, 29,034 leaf transcripts, 29,500 flower bud transcripts and 26,292 immature embryo transcripts were categorized, and ‘cellular process,’ ‘metabolic process,’ and ‘response to stimulus’ were the most highly represented terms. In cellular component, 29,387 stem transcripts, 31,707 root transcripts, 30,262 leaf transcripts, 30,866 flower bud transcripts, and 27,482 immature embryo transcripts were assigned, and ‘cell,’ ‘cell parts,’ and ‘organelle’ were prominently represented terms. In molecular function, 27,127 stem transcripts, 29,382 root transcripts, 28,083 leaf transcripts, 28,747 flower bud transcripts and 25,514 immature embryo transcripts were categorized, and ‘binding,’ ‘catalytic processes,’ and ‘transporter activity’ were the most dominantly groups. These dominated GO terms provided an overview of ontology content and indicated extensive biological activities being taking place in the five tissues of *B. napus*.

### Common Genes in Five Tissues of *B. napus*

**FIGURE 2:**
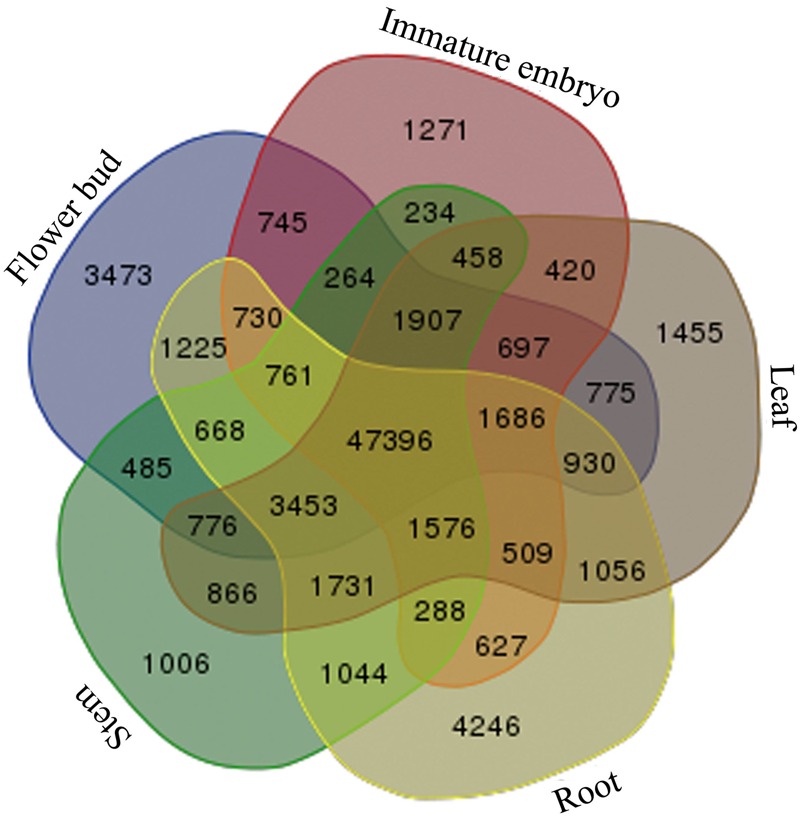
**Statistics of the common genes and tissue-specific genes.** Venn diagram was used for illustration of all 31 possible intersections between five independent transcriptome data sets including the number of genes composing each of these overlapping categories. Transcriptome data sets are as follows: the stems (light-green color), roots (light-yellow color), leaves (light-brown color), flower buds (light-blue color), and the immature embryos (light-purple color).

Afterward, to investigate common genes and tissue-specific genes, the transcripts of the five tissues were mutually compared, and a total of 47,396 common genes in the five tissues of *B. napus* were discovered, and 1,006 stem-specific genes, 4,246 root-specific genes, 1,455 leaf-specific genes, 3,473 flower bud-specific genes, and 1,271 immature embryo-specific genes were detected, respectively (**Figure 2**).

A total of 9,347 of 47,396 common genes were annotated by KEGG database and classified into 201 KEGG pathways. KEGG enrichment results showed that the top ten significantly enriched pathways (ranked by *p*-value) were ‘Ribosome’ (884 transcripts, *p*-value 7.93*E*-32, ko03010), ‘RNA transport’ (372 transcripts, *p*-value 1.77*E*-13, ko03013), ‘Splicesome’ (366 transcripts, *p*-value 8.05*E*-11, ko03040), ‘Proteasome’ (168 transcripts, *p*-value 1.49*E*-10, ko03050), ‘mRNA surveillance pathway’ (282 transcripts, *p*-value 6.51*E*-09, ko03015), ‘Ubiquitin mediated proteolysis’ (279 transcripts, *p*-value 9.38*E*-09, ko04120), ‘Protein processing in endoplasmic reticulum’ (386 transcripts, *p*-value 2.05*E*-08, ko04141), ‘Ribosome biogenesis in eukaryotes’ (212 transcripts, p-value 3.96*E*-07, ko03008), ‘Oxidative phosphorylation’ (413 transcripts, *p*-value 5.20*E*-07, ko00190), ‘RNA degradation’ (199 transcripts, *p*-value 9.22*E*-07, ko03018), (**Supplementary Figure 1**), it suggested that the common genes were dominantly involved in the basic metabolic pathways in *B. napus*.

**FIGURE 3:**
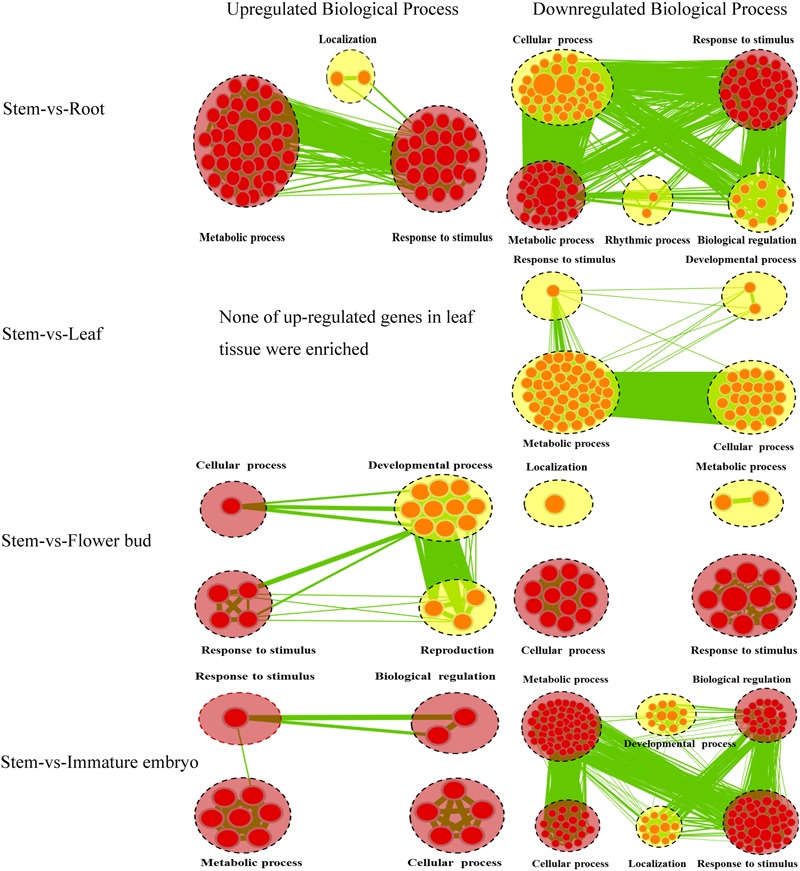
**Biological Process analysis of DEGs of the common genes between stems and other tissues.** GO modules enriched with up-regulated DEGs and down-regulated DEGs were visualized by the Enrichment Map in Cytoscape. The red and yellow circles represent the common and different biological processes between up-regulated and down-regulated DEGs, respectively. Clusters of functionally related gene-sets are manually circled with the black dashed ring, containing more red nodes which indicates this GO term contained more DEGs. Node size represents the gene-set size and edge thickness represents the degree of overlap between two gene-sets.

A lot of the common genes exhibited different expression levels in the five tissues, and therefore DEGs between stems and other tissues were identified using stem transcript expression levels as control. Between the stems and roots, 1,539 DEGs (*P* < 0.05) were detected, including 492 up-regulated and 1,047 down-regulated genes, and 1,024 of which could annotated by GO database (**Supplementary File 1**). We then performed Cytoscape Enrichment Map^4^ on GO classifications of these DEGs, and 884 DEGs between the stems and roots were assigned within the biological process category (**Figure 3**), and the common GO terms between up-regulated and down-regulated genes were ‘Metabolic process’ and ‘Response to stimulus.’ The up-regulated genes in roots were enriched in GO term of ‘Localization’ (28 transcripts). Among these genes, BnaA03g04410D homologous to AT5G13550 encoded sulfate transporter (SULTR) and was enriched in GO term of ‘anion transport.’ SULTR functioned in sulfate transmembrane transporter activity. SULTR2;1 in *Arabidopsis* roots was induced to increase uptake and root-to-shoot transport of sulfate under sulfur deficiency (Maruyama-Nakashita et al., 2015). The down-regulated genes were significantly enriched in ‘Cellular process’ (250 transcripts), ‘Biological regulation’ (87 transcripts) and ‘Rhythmic process’ (six transcripts). The down-regulated BnaA03g25300D homologous to AT4G04020 encoded fibrillin (FIB) and was enriched in GO term of ‘regulation photosynthesis.’ Plant fibrillins were a well-conserved protein family and performed a wide range of functions, such as abiotic stress tolerance, growth and development, hormone signaling, photoinhibition, etc (Singh and McNellis, 2011; Gámez-Arjona et al., 2014). SULTR up-regulation and FIB down-regulation in root tissue were very consistent with the biological functions of root tissues and supported the feasibility of GO classifications in **Figure 3**.

Between the stems and leaves, 278 DEGs (*P* < 0.05) (31 up-regulated and 247 down-regulated) were identified and 187 DEGs could be annotated by GO database (**Supplementary File 1**). A total of 139 DEGs between the stems and leaves were assigned in Biological process category and depicted a gene-set enrichment by Cytoscape Enrichment Map (**Figure 3**). None of up-regulated genes in leaves were enriched, and the down-regulated genes were enriched in GO terms of ‘Cellular process’ (53 transcripts), ‘Metabolic process’ (45 transcripts), ‘Response to stimulus’ (33 transcripts) and ‘Developmental process’ (17 transcripts). Among these genes, BnaA02g05780D homologous to AT5G22630 was enriched in GO term of ‘secondary metabolic process’ and encoded an arogenate dehydratase (ADT). ADT *in planta* functioned in the formation of phenylalanine (Phe) (Corea et al., 2012), that was the precursor for many secondary metabolites such as flavonoids, lignins, etc (Bross et al., 2011). ADT down-regulation in leaves and reversely up-regulation in stems was consistent with the biological functions of leaf tissues and stem tissues.

Between the stems and flower-buds, 860 DEGs (*P* < 0.05) (146 up-regulated and 714 down-regulated) were identified, 581 were annotated by GO database (**Supplementary File 1**), 478 were assigned in Biological process category and analyzed with Cytoscape Enrichment Map (**Figure 3**). The common GO terms between up-regulated and down-regulated genes were ‘Response to stimulus’ and ‘Cellular process.’ The up-regulated genes in flower-buds were enriched in ‘Developmental process’ (18 transcripts) and ‘Reproduction’ (10 transcripts). Among these genes, BnaA02g14990D homologous to AT1G70560 was enriched in GO term of ‘gynoecium development’ and encoded a tryptophan aminotransferase (TA). TA in *Arabidopsis* played an essential role in auxin biosynthesis and converted tryptophan to indole-3-pyruvate (IPA) (Won et al., 2011). TA up-regulation in flower-buds was consistent with the biological functions of flower-buds tissues. The down-regulated genes were enriched in ‘Metabolic process’ (119 transcripts) and ‘Localization’ (42 transcripts). The down-regulated BnaA05g30040D homologous to AT3G07570 was enriched in GO term of ‘histidine catabolic process’ and encoded a cytochrome b561/ferric reductase (FR). FR in *Arabidopsis* was reported to play a critical role in regulation of iron homeostasis (Xing et al., 2015).

Between the stems and immature embryos, 1,419 DEGs (*P* < 0.05) (121 up-regulated and 1,298 down-regulated) were identified and 978 were annotated by GO database (**Supplementary File 1**). A total of 745 DEGs between the stems and immature embryos were assigned in Biological Process with Cytoscape Enrichment Map (**Figure 3**), and the common GO terms between up-regulated genes and down-regulated genes were ‘Metabolic process,’ ‘Cellular process,’ ‘Biological regulation,’ and ‘Response to stimulus.’ The up-regulated genes in immature embryos were enriched in ‘Cellular process’ (26 transcripts), ‘Metabolic process’ (21 transcripts), ‘Response to stimulus’ (21 transcripts) and ‘Biological regulation’ (18 transcripts). Among these genes, BnaA02g00390D homologous to AT5G10160 was enriched in GO term of ‘Fatty acid biosynthetic process’ and encoded 3-hydroxyacyl-[acyl-carrier-protein (ACP)] dehydratase (HAD). HAD was a component of the type II fatty acid synthase complex involved in fatty acid biosynthesis in plants (González-Thuillier et al., 2016). Two genes encoding HAD in *Arabidopsis* were reported to highly express during lipid biosynthesis in seed development (Schmid et al., 2005). HAD up-regulation in immature embryos was very consistent with the biological functions of immature embryos tissues. The down-regulated genes were enriched in ‘Developmental process’ (81 transcripts) and ‘Localization’ (68 transcripts). The down-regulated BnaA02g01460D homologous to AT5G13180 was enriched in Go term of ‘xylem development’ and encoded NAC domain containing protein. The *Arabidopsis* NAC domain transcription factor VASCULAR-RELATED NAC-DOMAIN7 (VND7) was reported to act as a master regulator of xylem vessel differentiation (Yamaguchi et al., 2010). Down-regulation of NAC domain transcription factor in immature embryos and, however, up-regulation of which in stems were consistent with the biological functions of immature embryos and stem tissues.

Overall, the number of DEGs within the stems and roots (1,539 DEGs) was greater than that within the stems and immature embryos (1,419 DEGs), the stems and flower-buds (860 DEGs), the stems and leaves (278 DEGs), suggesting that the stems had more similar biological activities with the leaves than other tissues. ‘Response to stimulus’ was a common GO term between the stems and other four tissues, indicating that the response of a cell or an organism to a stimulus was all the processes that occurred in the five tissues.

**FIGURE 4:**
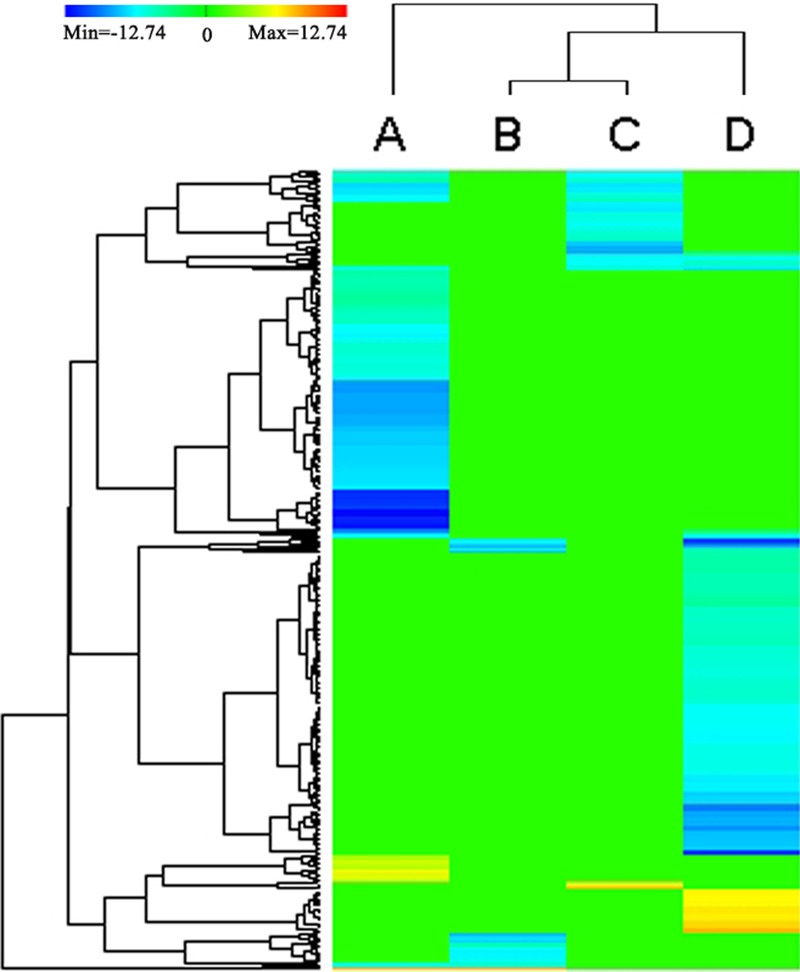
**Heat map depicting the DEGs (*P* < 0.05) annotated by KEGG databases.** Clustering of selected out genes expression profiles at 4 different comparisons. A, B, C, D represent the DEGs between the stems and roots, between the stems and leaves, between the stems and flower buds, between the stems and immature embryos, respectively. The color key represents RPKM (reads per kilobase per million mapped reads) normalized log2 transformed counts. Red color represents increasing level of the gene expression and blue color indicates decreasing of the gene expression after challenging with stem control. Green color represents no difference in gene expression. Each row represents a gene.

Kyoto Encyclopedia of Genes and Genomes enrichment analysis was also performed to further reveal the biological pathway of the DEGs between the stems and other tissues (**Figure 4**; **Supplementary File 2**). The DEGs (*P* < 0.05) between the stems and roots were dominated in the top three KEGG enrichment pathways (ranked by *p*-value), including ‘Photosynthesis’ (82 transcripts, *p*-value 6.80*E*-198, ko00195), ‘Photosynthesis-antenna proteins’ (19 transcripts, *p*-value 2.17*E*-14, ko00196) and ‘Carbon fixation in photosynthetic organisms’ (25 transcripts, *p*-value 1.72*E*-11, ko00710). Meanwhile, the DEGs between the stems and leaves were enriched in the top three KEGG enrichment pathways (ranked by *p*-value): ‘Cysteine and methionine metabolism’ (four transcripts, *p*-value 4.20*E*-03, ko00270), ‘Pentose phosphate pathway’ (three transcripts, *p*-value 7.35*E*-02, ko00030) and ‘Glycine, serine, and threonine metabolism’ (three transcripts, *p*-value 7.35*E*-03, ko00260). The DEGs between the stems and flower-buds were enriched in the top three KEGG enrichment pathways (ranked by *p*-value), e.g., ‘Cutin, suberine, and wax biosynthesis’ (46 transcripts, *p*-value 3.05*E*-05, ko00073), ‘Monoterpenoid biosynthesis’ (two transcripts, *p*-value3.11*E*-03, ko00902) and ‘Photosynthesis’ (eight transcripts, *p*-value 4.14*E*-03, ko00195). The DEGs between the stems and immature embryos were enriched in the top three KEGG enrichment pathways (ranked by *p*-value): ‘Phenylpropanoid biosynthesis’ (15 transcripts, *p*-value 2.12*E*-05, ko00940), ‘Plant hormone signal transduction’ (29 transcripts, *p*-value 2.5*E*-05, ko04075) and ‘Starch and sucrose metabolism’ (17 transcripts, *p*-value 7.04*E*-05, ko00500).

Except between the stems and leaves, the DEGs between the stems and roots/flower-buds/immature embryos shared a common KEGG pathway of ‘Cutin, suberine and wax biosynthesis (ko00073).’ In ‘Cutin, suberine, and wax biosynthesis’ pathway, the two DEGs (BnaA10g00380D and BnaC05g00460D) encoding putative CYP86A4 were down-regulated in the roots/flower buds/immature embryos and up-regulated in stems. CYP86A4 was required for the accumulation of C16 cutin monomers (Li-Beisson et al., 2009), and up-regulated expression of CYP86A4 could increase the synthesis of C16 cutin monomers, which was in accordance with increased synthesis of cutin in the stem.

**FIGURE 5:**
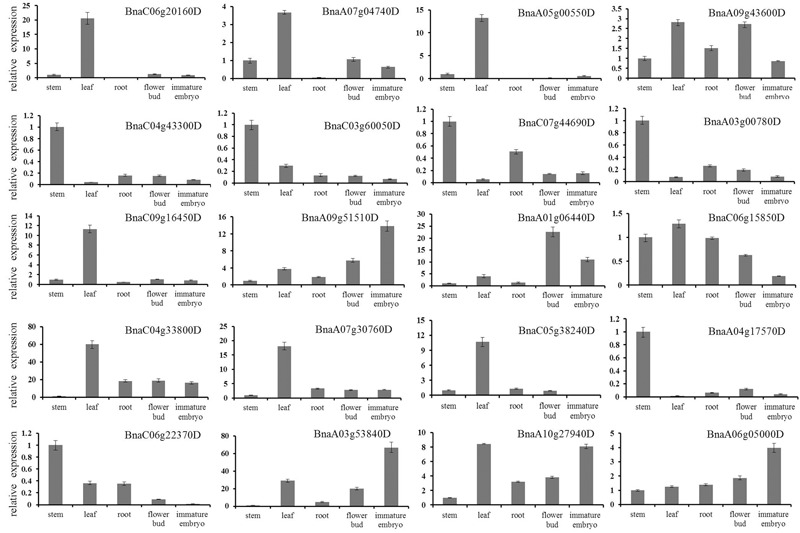
**Verification of differentially expressed genes by qRT-PCR.** Twenty DEGs were randomly chosen for qRT-PCR validation using stem transcript expression levels as control. The relative expression levels of each genes were expressed as the fold change between stem and other tissues. The *B. napus ACT 7 actin* gene was used as an internal control to normalize the expression data. The bars represent the standard deviation (*n* = 3).

Furthermore, we randomly selected 20 DEGs for qRT-PCR validation (**Figure 5**). The qRT-PCR profiles of 18 DEGs were basically in agreement with the RNA-seq results, for example, the expression levels of BnaC04g43300D and BnaC07g44690 were down-regulated in roots, leaves, flower buds and immature embryos in comparison to that in stems, although the expression fold change of them differed a little between the RNA-seq and qRT-PCR. However, the qRT-PCR profiles of two DEGs were not in agreement with the RNA-seq results: BnaC09g16450 was up-regulated in roots and immature embryos in the RNA-seq results, nevertheless down-regulated in qRT-PCR results; BnaC50g38240 was down-regulated in roots and leaves in the RNA-seq results, nevertheless up-regulated in qRT-PCR results.

### Tissue-Specific Genes in Five Tissues of *B. napus*

Tissue-specific genes were a group of genes of which function and expression were preferred in one or several tissues, and identification of tissue-specific genes would help for a better understanding of tissue-gene relationship, etiology and discovery of novel tissue specific targets (Xiao et al., 2010). A total, 1,006 stem-specific genes, 4,246 root-specific genes, 1,455 leaf-specific genes, 3,473 flower bud-specific genes, and 1,271 immature embryo-specific genes were detected. GO enrichment analysis (**Supplementary File 3**) showed that the top three enriched GO terms of the stem-specific genes were ‘Cellulase activity,’ ‘Plant-type cell wall modification,’ and ‘UDP-glucuronate decarboxylase activity’; the top three enriched GO terms of the root-specific genes were ‘Response to nitrate,’ ‘Nitrate transport,’ and ‘Transition metal ion transport’; the top enriched three GO terms of leaf-specific genes were ‘Response to auxin stimulus,’ ‘Electron carrier activity,’ and ‘Extracellular region’; the top three enriched GO terms of flower bud-specific genes were ‘Pollen exine formation,’ ‘Pollen wall assembly,’ and ‘Cellular component assembly involved in morphogenesis’; and the top enriched three GO terms of immature embryo-specific genes were ‘Nutrient reservoir activity,’ ‘Terpenoid biosynthetic process,’ and ‘Seed maturation.’ Overall, little overlap was observed for the top three enriched GO terms of the tissue-specific genes in the five tissues, demonstrating that various specific biological activities in the five tissues.

**FIGURE 6:**
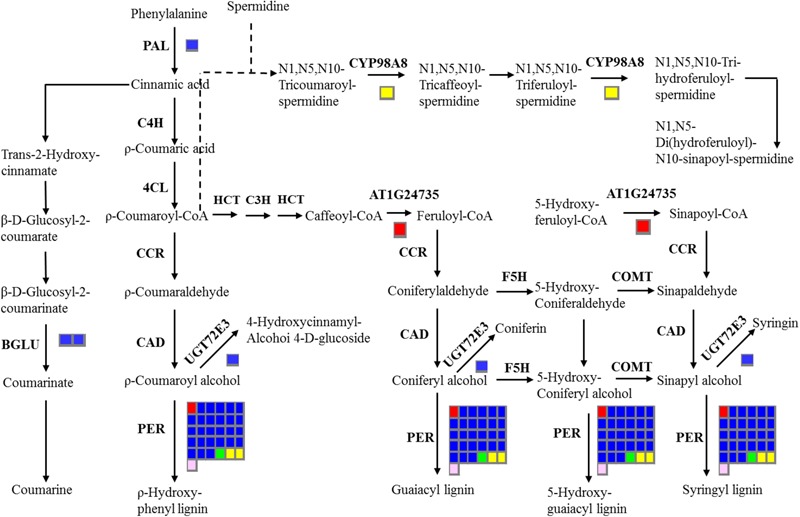
**Overview of the distribution of tissue-specific genes in ‘Phenylpropanoid biosynthesis’ pathway.** The number of genes in families with at least one gene differentially expressed among five tissues are indicated by the number of rectangles. The red, blue, green, yellow, and pink indicate the stem, root, leaf, flower bud, and immature embryo, respectively. Indentified enzymes include: CCoAOMT 1, Caffeoyl coenzyme A *O*-methyltransferase 1, AT4G34050; PER, Peroxidase; PAL3, Phenylalanine ammonia-lyase 3, AT5G04230; BGLU 45, beta glucosidase 45, AT1G61810; BGLU46, beta glucosidase 46, AT1G61820; UGT72E3, UDPG: coniferyl alcohol glucosyltransferase, AT5G26310; CYP98A8, Cytochrome P450, Family 98, Subfamily A, AT1G74540.

In addition, 16 stem-specific genes, 198 root-specific genes, 39 leaf-specific genes, 157 flower bud-specific genes, and 46 immature embryo-specific genes were annotated with KEGG databases (**Supplementary File 4**). The largest number of stem-specific genes (six members), leaf-specific genes (12 members), and immature embryo-specific genes (six members) were classified into ‘Plant hormone signal transduction’ pathway. The largest number of root-specific genes (59 members) were categorized in ‘Phenylpropanoid biosynthesis’ pathway, and the largest number of flower bud-specific gene were in ‘Pentose and glucuronate inter conversions’ pathway (21 members) (**Supplementary File 4**). The common KEGG pathways of tissue-specific genes included ‘Phenylpropanoid biosynthesis,’ ‘Methane metabolism,’ ‘Phenylalanine metabolism,’ ‘Plant hormone signal transduction,’ and ‘Peroxisome.’ The metabolites of the phenylpropanoid pathways were crucial for oil quality, seed coat color and defense response of *B. napus* (Zhao and Meng, 2003; Xiao et al., 2007; Zhao et al., 2009; Qu et al., 2013). Therefore, we further investigated the distribution of tissue-specific genes in ‘Phenylpropanoid biosynthesis’ pathway (**Figure 6**), and detected two stem-specific genes (AT1G24735, BnaAnng06470D; PER, BnaC09g19870D), five root-specific genes (PAL3, BnaA04g11940D; BGLU45, BnaA01g22890D; BGLU46, BnaA01g36270D; UGT72E3, BnaC07g16620D; PER, BnaA09g07380D), one leaf-specific gene (PER, BnaAnng05140D), two flower bud-specific genes (CYP98A8, BnaC06g22910D; PER, BnaA01g17520D) and one immature embryo-specific gene (PER, BnaA01g08600D) in this pathway. The tissue-specific genes in this pathway for all of the five tissues of *B. napus* possessed the members of PER family.

Kyoto Encyclopedia of Genes and Genomes enrichment analysis (**Supplementary File 4**) further depicted tissue-specific pathways: the stem-specific genes were enriched (ranked by *p*-value) in ‘Plant hormone signal transduction’ pathway (six transcripts, *p*-value 1.85*E*-04, ko04075); the root-specific genes were enriched in ‘Methane metabolism’ (56 transcripts, *p*-value 1.77*E*-104, ko00680), ‘Phenylpropanoid biosynthesis’ (59 transcripts, *p*-value 3.29*E*-51, ko00360) and ‘Phenylalanine metabolism’ (57 transcripts, *p*-value 1.43*E*-51, ko00940); the leaf-specific genes were enriched in ‘Plant hormone signal transduction’ pathway (12 transcripts, *p*-value 1.27*E*-06, ko04075); the flower bud-specific genes were enriched in ‘Pentose and glucuronate interconversions’ (21 transcripts, *p*-value 2.61*E*-14, ko00040), ‘Starch and sucrose metabolism’ (18 transcripts, *p*-value 4.27*E*-07, ko00500) and ‘Fatty acid elongation’ (five transcripts, *p*-value 9.42*E*-04, ko00062); the immature embryo-specific genes were enriched in ‘Monoterpenoid biosynthesis’ (two transcripts, *p*-value 3.09*E*-04, ko00902) pathway.

### Transcription Factors Identification and Network Analysis of the Transcripts

Transcription factors play an important role in regulating almost each aspect of the organism’s life metabolism (Wu et al., 2015). Plants devoted a large portion of their genome capacity to transcription, such as more than 1500 TFs in the *Arabidopsis* genome (Riechmann et al., 2000). By searching against the plant transcription factor database, 4,281, 4590, 4281, 4507, and 4017 potential TFs (*e*-value ≤ 1*E*-10) were detected in the transcriptomes of stems, roots, leaves, flower buds, and immature embryos, respectively. Among all of the TF transcripts, 3,410 of TF transcripts were common in the five tissues (**Supplementary Figure 2**), and the top three common TF families (ranked by transcript numbers) included ‘bHLH’ (1,409 transcripts), ‘NAC’ (1,008 transcripts), ‘MYB’ (916 transcripts). KEGG enrichment analysis showed that the common TF transcripts which were enriched in the top five pathway (ranked by *p*-value) was ‘Ribosome’ (22 transcripts, *p*-value 7.96*E*-14, ko03010), ‘Starch and sucrose metabolism’ (12 transcripts, *p*-value 7.77*E*-8, ko00500), ‘Amino sugar and nucleotide sugar metabolism’ (nine transcripts, *p*-value 8.25*E*-7, ko00520), ‘Aminoacyl-tRNA biosynthesis’ (13 transcripts, *p*-value 5.70*E*-5, ko00970) and ‘Phenylpropanoid biosynthesis’ (six transcripts, *p*-value 9.00*E*-5, ko00940) (**Supplementary File 5**), suggesting that the common TFs dominantly functioned in the regulation of the basic metabolic pathways.

Additionally, 27 stem-specific TFs, 187 root-specific TFs, 27 leaf-specific TFs, 156 flower bud-specific TFs, and 40 immature embryo-specific TFs (*e*-value ≤ 1*E*-10) were identified (**Supplementary Figure 2**). KEGG enrichment analysis results showed that two flower bud-specific TFs (fgenesh2_pg.C_Chr_05000117; GRMZM2G126957_P02) were enriched in two pathways (Aminoacyl-tRNA biosynthesis, ko00970; Protein processing in endoplasmic reticulum, ko04141), one root-specific TF (MDP0000291883) was enriched in ‘Plant–pathogen interaction’ (ko04626) pathway; however, none of immature embryo-specific, leaf-specific, and stem-specific TFs were enriched. Taken together, KEGG enrichment analysis of the common TFs and tissue-specific TFs suggested that the common TF families dominating maximal transcripts might play a more important role in biological and metabolic pathways of *B. napus* than the tissue-specific TFs.

**FIGURE 7:**
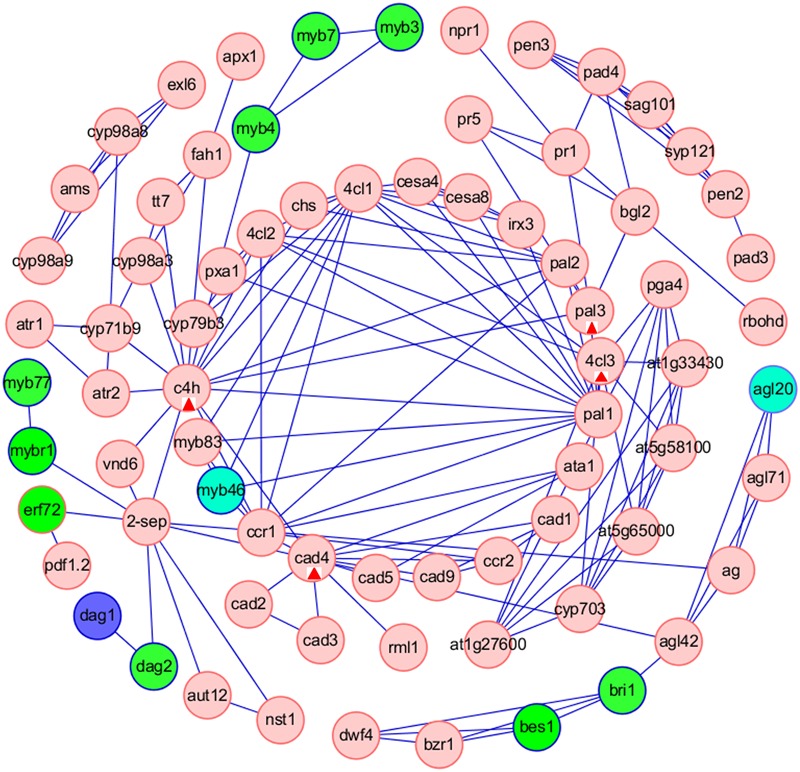
**Construction of TFs-based regulation networks about Phenylpropanoid biosynthesis pathway by Cytoscape software.** Green and red circles represent identified TFs and genes involved in the network, respectively. Compared with stem as control, the blue circles represent up-regulated TFs in root. The sky blue ellipses (MYB46) represent down-regulated TFs in leaf and immature embryos. The sky blue ellipses (AGL20) represent down-regulated TFs in root, flower bud and immature embryos. The red triangles label four nodes (C4H, PAL3, 4CL3, and CAD4) related to the ‘Phenylpropanoid biosynthesis’ pathway for other TF regulation.

Furthermore, we analyzed the interaction between TFs and the target genes involved in ‘Phenylpropanoid biosynthesis’ pathway, that was one of the common pathways of tissue-specific genes in the five tissues. We used the Cytoscape software to construct a TF-based regulation network with 110 nodes and 195 edges (**Figure 7**), and a total of 12 TFs, including MYB46, MYB4, MYB3, MYB7, EFR72, MYBR1, MYB77, DAG1, DAG2, BES1, BRI1, AGL20, were involved in the regulation network. Network analysis showed that ERF72, MYBR1, and DAG2 might directly interact with ‘2-SEP,’ or interact with ‘2-SEP’ and then ‘VND6,’ and further interact with C4H to function on ‘Phenylpropanoid biosynthesis’ pathway, implying that a novel gene similar as ‘*2-sep* (stress enhanced protein 2)’ existed in *B. napus*. Network analysis also showed that AMS might firstly interact with several targets, such as CYP98A8, CYP71B9, and then interact with C4H to function on the ‘Phenylpropanoid biosynthesis’ pathway. AMS (Aborted Microspores) was reported as a master regulator coordinating pollen wall development and sporopollenin biosynthesis in *A. thaliana*, and CYP98A8, as one of AMS targets was required for the production of phenolic precursors (Xu et al., 2014). Moreover, we observed that MYB3, MYB7 might interact with MYB4 to regulate the C4H in the ‘Phenylpropanoid biosynthesis’ pathway. These results showed that C4H, the gene of that was one of common phenylpropanoid biosynthetic genes (Qu et al., 2013), might be a key node related to the ‘Phenylpropanoid biosynthesis’ pathway in *B. napus*. In addition, Network analysis showed that PAL3, 4CL3, and CAD4 might be nodes related to the ‘Phenylpropanoid biosynthesis’ pathway for other TF regulation.

## Discussion

RNA-seq technology is an effective way to analyze the expression of individual transcripts in any one species (Parkin et al., 2010) and which has been widely used for analyzing gene expression patterns in different tissues and different developmental stages under various conditions (Geng et al., 2011). In the present study, the RNA-seq on the stem transcriptome of ZY821 at initial flowering stage was conducted and 134,015,130 clean reads with 100 bp length were generated, more than 90% of which had perfect match to the reference genome.

Given that there were available transcriptome sequencing data of other tissues in *B. napus*, we therefore downloaded the transcriptome data of roots, leaves, flower buds, and immature embryos to compare with our stem transcriptome for different gene expression analysis. The downloaded root transcriptome was from *B. napus* N119, that was used for investigation of a mechanism that respond to sudden increase in salinity (Yong et al., 2014). The downloaded leaf transcriptome was from Ningyou 7, which and other four natural *B. napus* leaf transcriptome (Tapidor, Altasweet, Ceska, and Aphid resistant rape) were investigated by RNA-seq to quantify transcript abundance in polyploids and estimate the relative contributions of homoeologous genes (Higgins et al., 2012). The compared flower bud transcriptome was from *B. napus* H3, which and the flower bud transcriptome of the female sterile disomic addition line (S1) were investigated by RNA-seq to reveal the metabolic pathways behind the formation of the damaged gynoecium (Fu et al., 2014). The compared immature embryos transcriptome was from *B. napus* CR 3170. Transcriptome analysis of cultured immature embryos of *B. napus* CR 3170 and 3231, which contrast for seed lipid accumulation, were performed to relate transcript abundance with metabolic flux (Schwender et al., 2014).

In the present study, the five tissue transcriptome were from different cultivars. Although there were differences among the expression of these different tissues, comparison of downloaded transcriptomes of different tissues also could reveal some interesting results, for example, Wu et al. (2015) combined leaf sequencing data with downloaded root sequencing data to obtain the better transcriptome assembly of *Raphanus sativus*, and a total of 3,563 DEGs and tissue-specific biological processes between leaf and root tissues were detected, this study was commented as ‘not only provide an important genomic resource for *R. sativus*, but also will facilitate future network-based functional genomic analyses and will provide insights into the systematic analysis of high-throughput sequencing data’ (Gu and Lu, 2016); Ramsköld et al. (2009) characterized the features of mammalian tissue transcriptomes through comparative analysis of the downloaded RNA-Seq data, including human tissues, mouse tissues, mouse embryonic cell and body data, cerebellum data from non-schizophrenic humans, and revealed about 8,000 genes were ubiquitously expressed in most tissues to support a model in which variable exterior components feed into a large, densely connected core composed of ubiquitously expressed intracellular proteins. In addition, in order to reduce the cultivar difference, all of the raw reads from the five tissues in the present study were filtered, assembled, mapped to the reference genome, annotated by GO and KEGG databases, and identification of common genes, DEGs and tissue-specific genes was performed, using the same bioinformatics software with the same parameters. The reads from the five tissues were great different, such as 134,015,130 reads from stem, and, however, 6,945,504 reads from flower bud. In order to accurate the DEGs basing on these data, we used DESeq2 software to test the DEGs between stems and other tissues, because DESeq used a normalization factor within the statistical model, that based on a negative binomial distribution and local regression, for differential analysis, rather than on the data themselves (Dillies et al., 2013). DESeq relied on the hypothesis that most of the genes were not different expression, and DESeq performed much better than other normalization methods [Such as Total Count (TC), Upper Quartile (UQ), Median (Med), Quantile (Q), and the RPKM normalization] for data with differences in library composition (Dillies et al., 2013). In the meanwhile, we randomly selected 20 DEGs for qRT-PCR validation and 18 DEGs were basically in agreement with the downloaded RNA-seq results, which support that the present transcriptomes comparison between different tissues could be reliable in some degree. Under the current condition that there were no transcriptome sequencing data of different tissues from the same *B. napus* ZY821 cultivar, comparison of the stem transcriptome of ZY821 with available tissue transcriptome data of other cultivars could provide some valuable enlightenments for further research of different tissues and also could provide the common and specific unigenes in different tissues in some degree.

The common genes in the five tissues of *B. napus* were dominantly involved in the basic metabolic pathway, such as ‘Ribosome,’ ‘RNA transport,’ ‘Splicesome,’ ‘Proteasome,’ ‘mRNA surveillance pathway.’ ‘Ribosome’ was involved in the final stage of gene expression, and Ribosome biogenesis was a key point for the regulation of cell growth and division (Dez and Tollervey, 2004). ‘RNA transport’ from the nucleus to the cytoplasm was fundamental for gene expression, and nuclear export of mRNAs was functionally coupled to different steps in gene expression processes, such as transcription, splicing, etc. ‘Spliceosome’ functioned in the following splicing on excise introns and join exons of eukaryotic mRNA precursors after transcription. ‘Proteasome’ was a protein-destroying apparatus involved in many essential cellular functions, such as regulation of cell cycle, cell differentiation, signal transduction, stress signaling. ‘mRNA surveillance pathway’ was a quality control mechanism that detected and degraded abnormal mRNAs.

A relatively small portion of DEGs showed similar biological activities between two tissues. The number of DEGs within the stems and leaves (278 DEGs) was minimum, and none of up-regulated DEGs in leaves when compared to stems were enriched within the biological process category, suggesting that the stems and leaves had similar biological activities. The stem-specific genes were dominantly related to plant disease resistance and stress responses, such as the enriched GO terms of ‘Plant-type cell wall modification,’ ‘UDP-glucuronate decarboxylase activity.’ The leaf-specific genes were remarkably involved in plant stimulus responses and hormone signal, such as the GO terms of ‘Response to auxin stimulus’ and ‘Electron carrier activity.’ In addition, both of leaf-specific genes and stem-specific genes were enriched in the KEGG pathway of ‘Plant hormone signal transduction.’ ‘Plant hormone signal transduction’ pathway was reported to take positive action in stems of ZY821 after infected by *S. sclerotiorum* (Zhao et al., 2009), and also was reported to relate to stem swelling of *B. juncea* (Sun et al., 2012), and also was found in the salt-responsive transcriptome in the leaves in *B. napus* (Yong et al., 2014).

The up-regulated DEGs in roots and root-specific genes were closely related to material transportation and stress responses, such as root-specific genes enriched GO terms of ‘Nitrate transport,’ ‘Transition metal ion transport,’ and DEGs-enriched GO term of ‘Localization’ and ‘Response to stimulus.’ ‘Response to stimulus’ was reported as one of overrepresented GO terms in up-regulated DEGs in *B. napus* N119 roots after salinity stress (Yong et al., 2014). The up-regulated DEGs in flower buds and flower bud-specific genes were obviously involved in flower development, such as flower bud-specific genes enriched GO terms of ‘Pollen exine formation,’ ‘Pollen wall assembly,’ and DEGs enriched GO term of ‘Developmental process.’ Similar observation has been reported in the flower bud transcriptome of *B. napus* H3, and many dominant genes in the flower buds were involved in the development of gynoecium, embryo sac, as well as the interactions between pollen and pistil (Fu et al., 2014). Meanwhile, our analysis showed that the up-regulated DEGs in immature embryos and immature embryo-specific genes were dominantly related to seed development such as DEGs-enriched GO term of ‘Biological regulation,’ and immature embryo-specific genes-enriched GO terms of ‘Nutrient reservoir activity’ and ‘Seed maturation.’

Regulation of gene expression at the level of transcription influenced or controlled many of the biological processes in a cell or organism (Riechmann et al., 2000). *Arabidopsis* dedicates over 5% of its genome to code for more than 1500 TFs (Riechmann et al., 2000). In the present study, 4,281, 4590, 4281, 4507, and 4017 potential TFs were detected in the transcriptomes of stems, roots, leaves, flower buds and immature embryos, respectively, occupying 3.9–4.5% of the total genes in the *B. napus* reference genome, that contained 101,040 gene models (Chalhoub et al., 2014). The number of TFs in *B. napus* was greater than that of *Arabidopsis*, the reason for which was that the genome of allotetraploid *B. napus* (2*n* = 38) containing 38 chromosomes was more capacity than that of diploid *Arabidopsis thaliana* (2*n* = 10) containing 10 chromosomes.

The top three TF families of the common TF transcripts in the five tissues were ‘bHLH,’ ‘NAC,’ and ‘MYB.’ The bHLH (basic helix-loop-helix) TFs participated in controlling cell proliferation and development of specific cell lineages (Heim et al., 2003). The NAC TFs were multifunctional proteins with various roles in the plant life cycle (Nuruzzaman et al., 2010), such as maintenance of the shoot apical meristem (Kim et al., 2007), lateral root development (He et al., 2005), hormone signaling (Greve et al., 2003), response to pathogen infection (Olsen et al., 2005), embryo development (Duval et al., 2002). The MYB TFs were associated with regulation of secondary metabolism, cellular morphogenesis, meristem formation, and cell cycle (Jin and Martin, 1999; Zhong et al., 2007). The common TF transcripts in the five tissues were dominantly involved in the regulation of the basic metabolic pathways, such as the enrichment pathways of ‘Ribosome,’ ‘Starch and sucrose metabolism,’ and ‘Amino sugar and nucleotide sugar metabolism.’ However, a small number of tissue-specific TFs were detected, and only a few tissue-specific TFs could be enriched in KEGG pathways. The common TF families might play a more important role than the tissue-specific TFs in biological activities and metabolic pathways of *B. napus*.

The TFs-based regulation network involved in ‘Phenylpropanoid biosynthesis’ pathway suggested that a novel gene similar as ‘*2-sep*’ and four key nodes (C4H, PAL3, 4CL3 and CAD4) might exist in *B. napus*. ‘2-SEP’ (GenBank no. 103642107) in *Zea* might act as one chloroplastic-like protein (Alexandrov et al., 2009) and ‘2-SEP’ (AT2G21970) in *A. thaliana* was as one potential light stress-regulated chlorophyll-binding protein (Heddad and Adamska, 2000). C4H (cinnamate-4-hydroxylase, AT2G30490) was reported to impact phenylpropanoid metabolism (Schilmiller et al., 2009). PAL3 (Phenylalanine ammonia-lyase 3, AT5G04230) was reported to differ from PAL1 and PAL2, and PAL3 promoter region lacked several motifs conserved between *A. thaliana* PAL1 and PAL2, as well as motifs described in other genes involved in phenylpropanoid metabolism (Wanner et al., 1995; Raes et al., 2003; Cochrane et al., 2004). 4CL3 (isoform of 4-coumarate:CoA ligase, AT1G65060) was involved in the last step of the general phenylpropanoid pathway and had a role in flavonoid biosynthesis (Ehlting et al., 1999). CAD4 (cinnamyl alcohol dehydrogenase 4, AT3G19450) was reported to couple with CAD5 to function in lignin biosynthesis (Sibout et al., 2003, 2005; Tronchet et al., 2010). In the meanwhile, Network analysis showed that MYB46 interacted with four targets, including MYB83 and CCR1, 4CL1, PAL1 (**Figure 7**). MYB46 (At5g12870) and its close homolog MYB83 (At3g08500) were master regulators of secondary wall formation in *Arabidopsis* (Zhong et al., 2007; Ko et al., 2012), and functioned redundantly in the transcriptional regulatory cascade leading to secondary wall formation in fibers and vessels (McCarthy et al., 2009). PAL1 (phenylalanine ammonia-lyase 1, At2g37040) and CCR1 (cinnamoyl-CoA reductase 1, At1g15950) were primary enzymes of *Arabidopsis* phenylpropanoid metabolism (Raes et al., 2003; Fraser and Chapple, 2011). CCR1 (At1g15950) and 4CL1 (4-coumarate:CoA ligase, At1g51680) were involved in lignin biosynthesis (Ehlting et al., 1999; Jones et al., 2001; Lauvergeat et al., 2001; Fraser and Chapple, 2011).

## Conclusion

The stem transcriptome of ZY821 at initial flowering stage was sequenced and approximately 13.4 Gb high-quality clean reads were obtained, which were used for comparative transcriptome analysis with the transcriptome of roots, leaves, flower buds, and immature embryos of *B. napus*. Accordingly, common genes, DEGs and tissue-specific genes in five tissues were detected, and dominantly biochemical activities and pathways involving these genes were depicted. A TFs-based regulation network involved in ‘Phenylpropanoid biosynthesis’ pathway was constructed to suggest a novel gene similar as ‘*2-sep*’ and four key nodes (C4H, PAL3, 4CL3, and CAD4) in *B. napus*. Taken together, this study provided a useful stem transcriptome resource and valuable comparative transcriptome information of five tissues of *B. napus* for future research.

## Author Contributions

LM and ML conceived and designed the experiments. NR performed the RNA isolation experiment. LM analyzed the data and wrote the manuscript. LZ, GL, and XZ helped to analyze the data and draft the manuscript. JX and JG participated in the design of the study. ML and CF coordinated the study and revised the manuscript. All authors read and approved the final manuscript.

## Conflict of Interest Statement

The authors declare that the research was conducted in the absence of any commercial or financial relationships that could be construed as a potential conflict of interest.

## Footnotes

1 http://www.genoscope.cns.fr/brassicanapus/

2 http://planttfdb.cbi.pku.edu.cn/download.php

3 http://www.genoscope.cns.fr/brassicanapus/

4 http://www.cytoscape.org/

## Supplementary Material

FIGURE 1 **KEGG enrichment analysis of the common genes**.

FIGURE 2 **Statistics of the common transcription factors and tissue-specific transcription factors**.

TABLE 1 **The primers for qRT-PCR validation of DEGs**.

TABLE 2 **The gene expression levels of all the transcripts**.

SUPPLEMENTARY FILE 1 **GO annotation of the DEGs of the common genes**.

SUPPLEMENTARY FILE 2 **KEGG enrichment analysis of the DEGs of the common genes between stem and other tissues**.

SUPPLEMENTARY FILE 3 **GO enrichment analysis of the tissue-specific genes**.

SUPPLEMENTARY FILE 4 **KEGG classification and enrichment analysis of the tissue-specific genes**.

SUPPLEMENTARY FILE 5 **KEGG enrichment analysis of the common transcription factors (Top 20 pathways)**.
