# Supplementary figures and images for: Transcriptome Analysis of Stem and Globally Comparison with Other Tissues in Brassica napus
Source: Front Plant Sci. 2016 Sep 21;7:1403. doi: 10.3389/fpls.2016.01403 (PMC5030298; doi:10.3389/fpls.2016.01403)

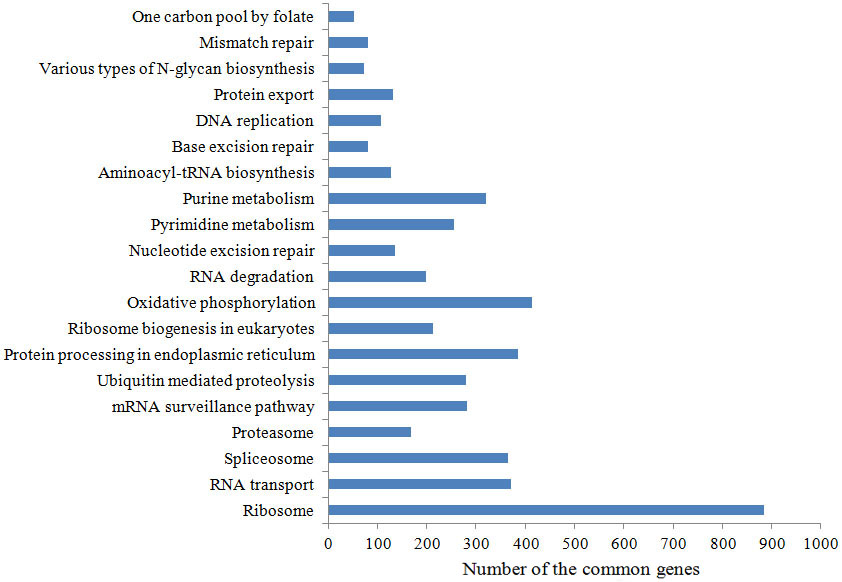

Supplement: FIGURE 1 — KEGG enrichment analysis of the common genes. [file Image_1.JPEG]

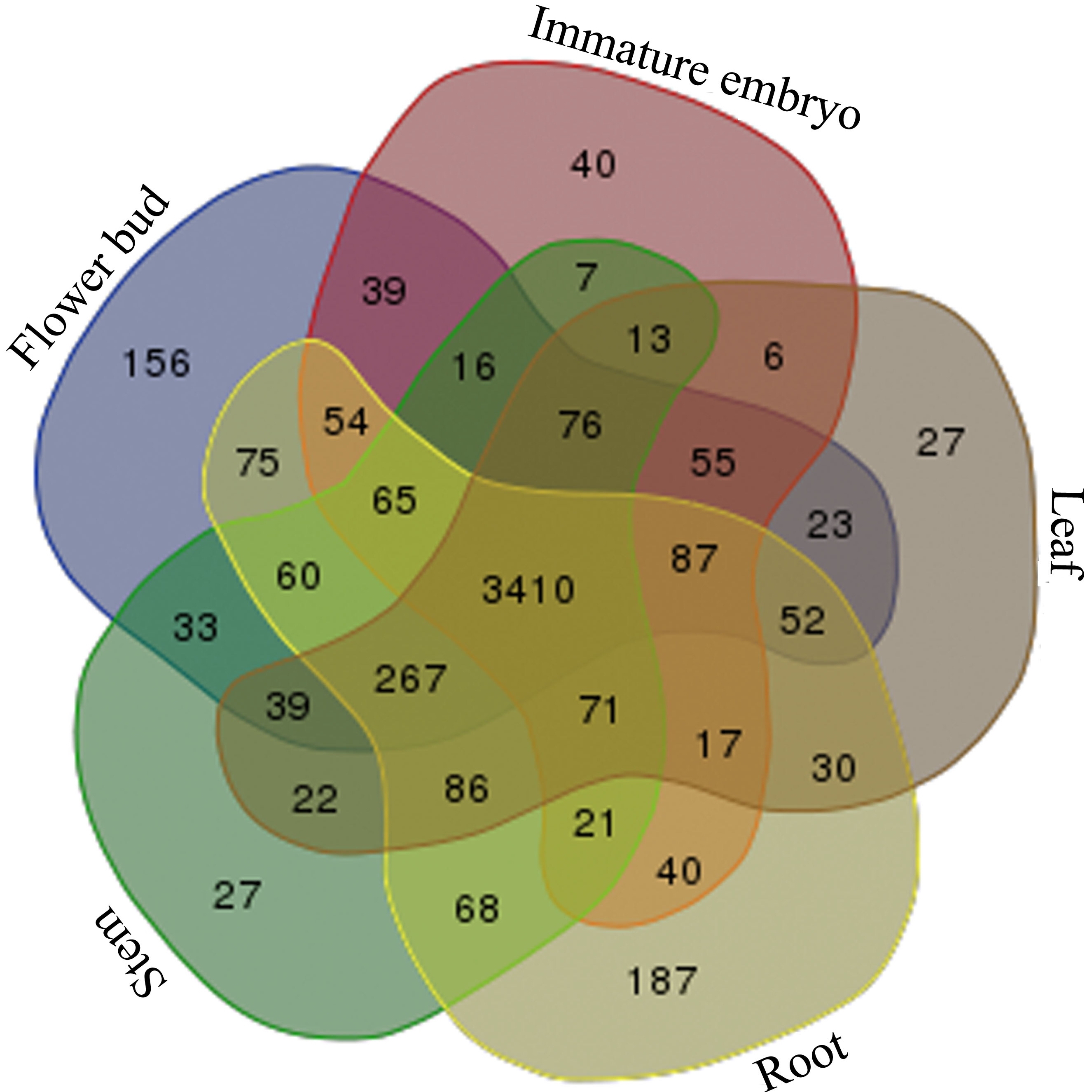

Supplement: FIGURE 2 — Statistics of the common transcription factors and tissue-specific transcription factors. [file Image_2.JPEG]
