# Supplementary material for: Transcriptome Analysis of Stem and Globally Comparison with Other Tissues in Brassica napus
Source: Front Plant Sci. 2016 Sep 21;7:1403. doi: 10.3389/fpls.2016.01403 (PMC5030298; doi:10.3389/fpls.2016.01403)
Supplement: TABLE 1 — The primers for qRT-PCR validation of DEGs. [file Table_1.DOC]

**Supplement Table 1. The primes for qRT-PCR validation of DEGs.**

| Gene | Primes |
| --- | --- |
| Actin | Forward: 5’- CCCTGGAATTGCTGACCGTA-3’  Reverse: 5’- TGGAAAGTGCTGAGGGATGC-3’ |
| BnaC06g20160D | Forward: 5’- AGGATTGCCCTCGCTCACA -3’  Reverse: 5’- CCTCAAGTCCATGCTGCCGTT -3’ |
| BnaA07g04740D | Forward: 5’- ACCGTTCAGCACCAATCTACACA -3’  Reverse: 5’- TTGATAACACAACCTTCACCGAT -3’ |
| BnaA05g00550D | Forward: 5’- CTATCTGCCCCTGTTCGCTTG -3’  Reverse: 5’- TCTCCTTCCGATGTCGCCTT -3’ |
| BnaA09g43600D | Forward: 5’- TCTGGAAACAGCCCACGAAC -3’  Reverse: 5’- TGAAGAGCTTGTATCCACCGTA-3’ |
| BnaC04g43300D | Forward: 5’- ACAATCACGCTCATGACTGCT-3’  Reverse: 5’- TCCTCAAAATCATCCGTTGC-3’ |
| BnaC03g60050D | Forward: 5’- AGTCTACGACAAGAAAGCACCC -3’  Reverse: 5’- ATCACATGACCACGCAAGCAG -3’ |
| BnaC07g44690D | Forward: 5’- ACACCAAGAAGTCGGTCACA-3’  Reverse: 5’- AACTGTCCTTCGTCTGCCGAT -3’ |
| BnaA03g00780D | Forward: 5’- GACCGATAACGCCTTTAGCAG -3’  Reverse: 5’- GGGAACTTACCGTTTTGTCCAT-3’ |
| BnaC09g16450D | Forward: 5’- TAACAGTAAACGGACCCGAAC -3’  Reverse: 5’- TCGGTGCCCCAATATCTGC -3’ |
| BnaA09g51510D | Forward: 5’- TAACAACGGACAGTTCTACGAG-3’  Reverse: 5’- AGACATCCCAATCGCCCACA-3’ |
| BnaA01g06440D | Forward: 5’- CGAATACCGACAGCACACGTT -3’  Reverse: 5’- TCAGAGCCTCGTCCATAGCAT -3’ |
| BnaC06g15850D | Forward: 5’- CTCAAAGGCTTCTCATTTTCGTT -3’  Reverse: 5’- CAAGACTCTATTGACGGCACCA-3’ |
| BnaC04g33800D | Forward: 5’-GGTTCGGCTTCATCACACC -3’  Reverse: 5’- GCAAGGCTACGATAACCCTCA-3’ |
| BnaA07g30760D | Forward: 5’-TCAACTTATTGGGACGGCAAC -3’  Reverse: 5’- TTTCACGAACACAACCTCGAA-3’ |
| BnaC05g38240D | Forward: 5’-GGAACCAGCTACACCTTCGAC -3’  Reverse: 5’- TCTTCTGCACCATTAACGTCCA-3’ |
| BnaA04g17570D | Forward: 5’-CAGCTTGCACATCCTAAACCAC -3’  Reverse: 5’-CCTTTACCGACACTCACAACCC-3’ |
| BnaC06g22370D | Forward: 5’-TGCTCCATCAATCAACGGACA -3’  Reverse: 5’-TAAAACACAACGGCACACCA -3’ |
| BnaA03g53840D | Forward: 5’- GCTAAAATCTTGCTCGAGACCA-3’  Reverse: 5’- TTCAAGCGTTCCTATCACCAG-3’ |
| BnaA10g27940D | Forward: 5’- AAACTCATACCACAGCCGTTC -3’  Reverse: 5’-ATACATCCCAGCGTCGGAGA -3’ |
| BnaA06g05000D | Forward: 5’-CGCACTCCAAGAGAACCTCC -3’  Reverse: 5’-GCTGCAACGTTCACTCTAATCCC -3’ |
