# Supplementary material for: Transcriptome Analysis of Stem and Globally Comparison with Other Tissues in Brassica napus
Source: Front Plant Sci. 2016 Sep 21;7:1403. doi: 10.3389/fpls.2016.01403 (PMC5030298; doi:10.3389/fpls.2016.01403)
Supplement: TABLE 2 — The gene expression levels of all the transcripts. [file Table_2.DOC]

**Supplement Table 2.** The gene expression levels of all the transcripts.

| Sample | Number of genes with RPKM values of 0-0.1 | Percentage of genes with RPKM values of 0-0.1 in the total genes* | Number of genes with RPKM values of 0.1-3.75 | Percent of genes with RPKM values of 0.1-3.75 in the total genes* | Number of genes with RPKM values of 3.75-15 | Percent of genes with RPKM values of 3.75-15 in the total genes* | Number of genes with RPKM values >15 | Percent of genes with RPKM values >15 in the total genes* |
| --- | --- | --- | --- | --- | --- | --- | --- | --- |
| Stems | 38,127 | 37.73% | 34,314 | 33.96% | 18,728 | 18.54% | 9,868 | 9.77% |
| Roots | 33,114 | 32.77% | 31,651 | 31.33% | 21,542 | 21.32% | 14,729 | 14.58% |
| Leaves | 39,203 | 38.80% | 33,114 | 32.78% | 18,171 | 17.98% | 10,543 | 10.44% |
| Flower buds | 35,069 | 34.71% | 25,050 | 24.79% | 20,896 | 20.68% | 20,014 | 19.81% |
| Immature embryos | 43,184 | 42.74% | 32,326 | 31.99% | 16,003 | 15.84% | 9,520 | 9.42% |

* “% value” represents the percentage of the expressed genes of the tissue transcriptome in the total genes of the *B. napus* reference genome, that contained 101,040 gene models ([Chalhoub, et al.,](http://www.ncbi.nlm.nih.gov/pubmed/?term=Chalhoub B%5BAuthor%5D&cauthor=true&cauthor_uid=25146293) 2014). The expressed genes with RPKM ≥0.1 of the tissue transcriptome were used for further annotation and comparison analysis in this study.
